# Supplementary material for: Construction of Whole Genomes from Scaffolds Using Single Cell Strand-Seq Data
Source: Int J Mol Sci. 2021 Mar 31;22(7):3617. doi: 10.3390/ijms22073617 (PMC8037727; doi:10.3390/ijms22073617)
Supplement: Supplementary file 1 [file ijms-22-03617-s001.zip › Hills et al - Supplementary Information/Supplementary Information Dec 24 VLPL.docx]

**Supplementary Figure 1**


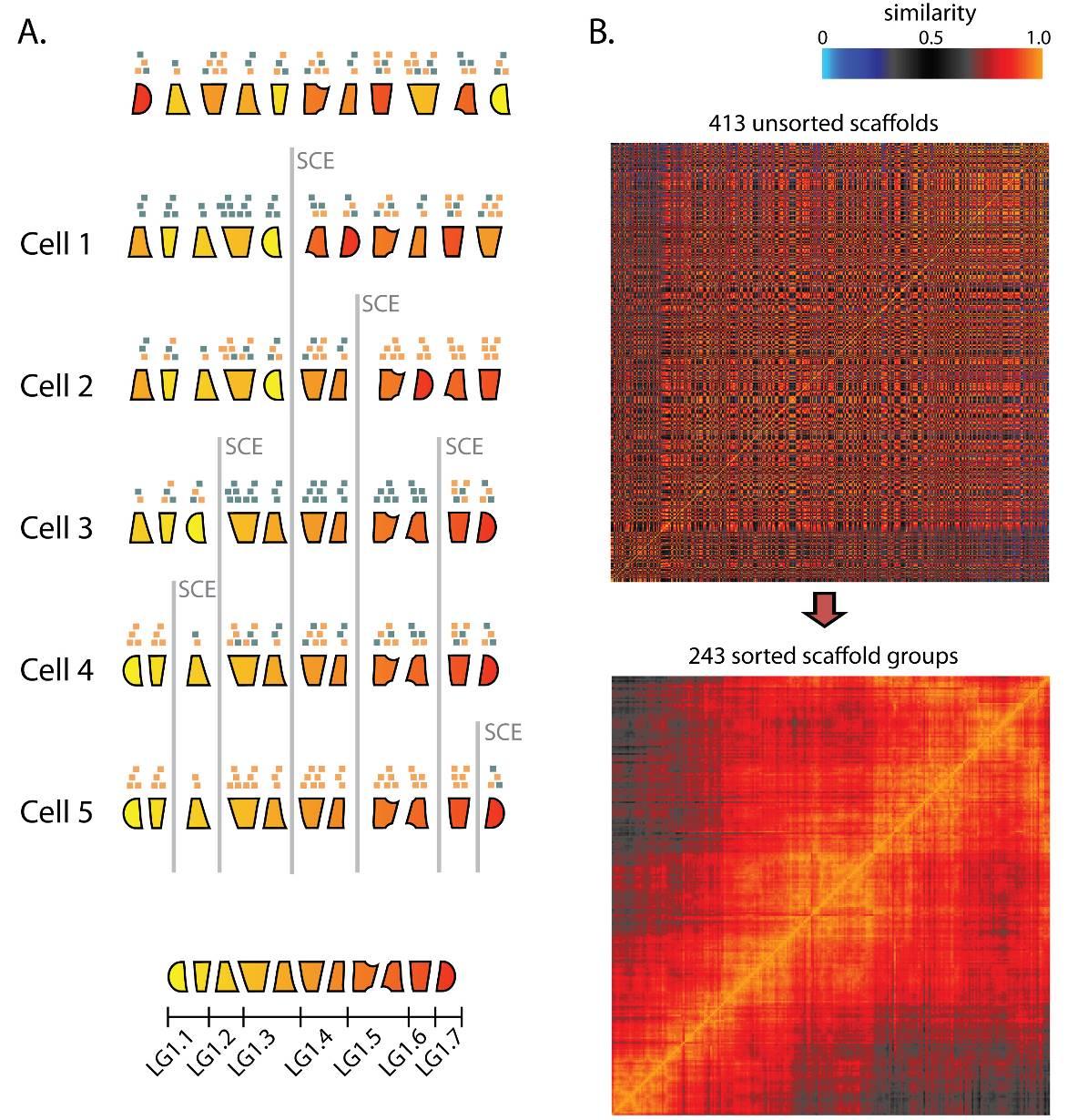


**Supplementary Figure 1: Clustering scaffolds within Linkage Groups.**

A. Schematic for clustering 11 scaffolds within Linkage Groups (LG). Across 5 different cells, SCEs reshuffle the pattern of template strands between scaffolds. Scaffolds are clustered into groups of similarity upstream and downstream of an SCE event. As more SCEs are included, more ordered sub-groups are resolved. Clustering is performed through a Monte Carlo algorithm. B. Example heat plots of ferret LG consisting of 413 scaffolds. While some scaffolds show identical template patterns (due to their close proximity), 243 scaffold groups can be elucidated and ordered. The greatest degree of template strand similarity is between nearby scaffolds, with progressively weakening linkage between more distant scaffolds.

**Supplementary Figure 2**


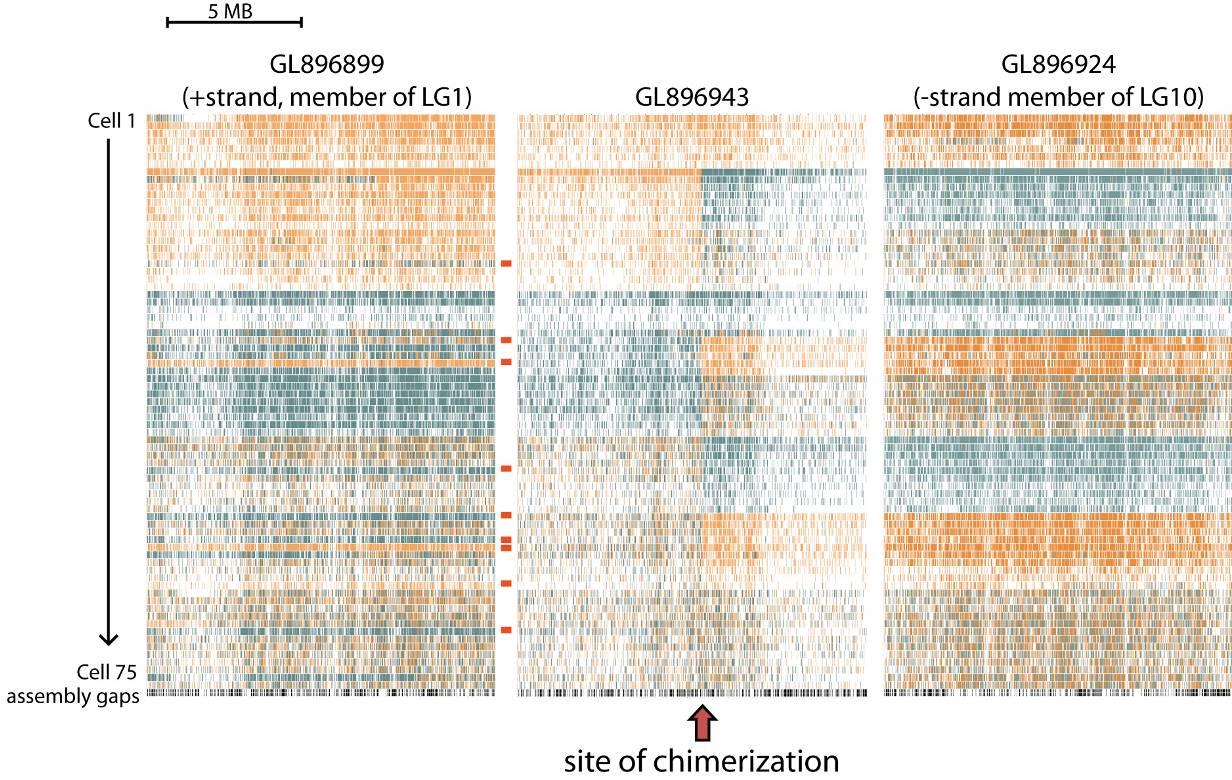


**Supplementary Figure 2: Example of chimeric scaffold in the ferret.**

GL896943, a ~12 MB scaffold in the ferret assembly, was flagged as a putative chimera due to a change in template strand state occurring at the same location in multiple cells (red arrow). Our software splits these scaffolds at the site of chimerization into two smaller contigs prior to clustering. In this example, one contig matched scaffolds belonging to LG1 (GL896899 used as a representative member), and the other matched scaffolds belonging to LG10 (GL896924 used as a representative member). Note the nine differences (red bars) between GL896899 and GL896943, occurred from SCEs between these fragments that are distantly located within the LG.

**Supplementary Figure 3**


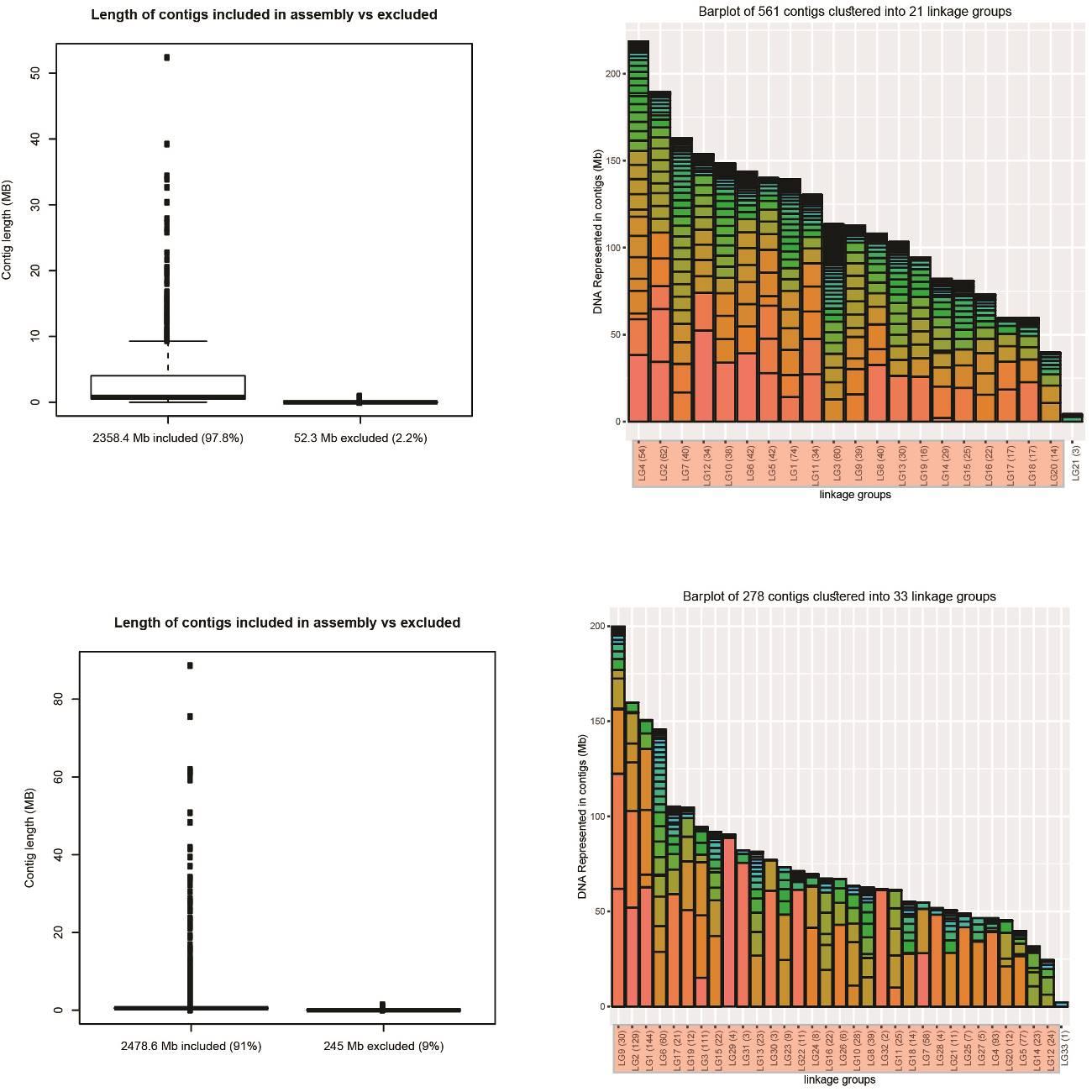


**Supplementary Figure 3: Scaffolds clustered into chromosome-sized linkage groups (LGs).**

The ferret (top panels) and Guinea pig (bottom panels) assemblies consist of thousands of scaffolds of no known location. Boxplots show that the majority of scaffolds are placed into LGs by Strand-seq analysis, with generally small fragments excluded (colors used to distinguish scaffold sizes). Bar plots show the distribution of scaffolds that have clustered together into LGs based on template inheritance patterns. LGs highlighted in red represent the expected number of chromosomes for that organism.

**Supplementary Figure 4:**

**
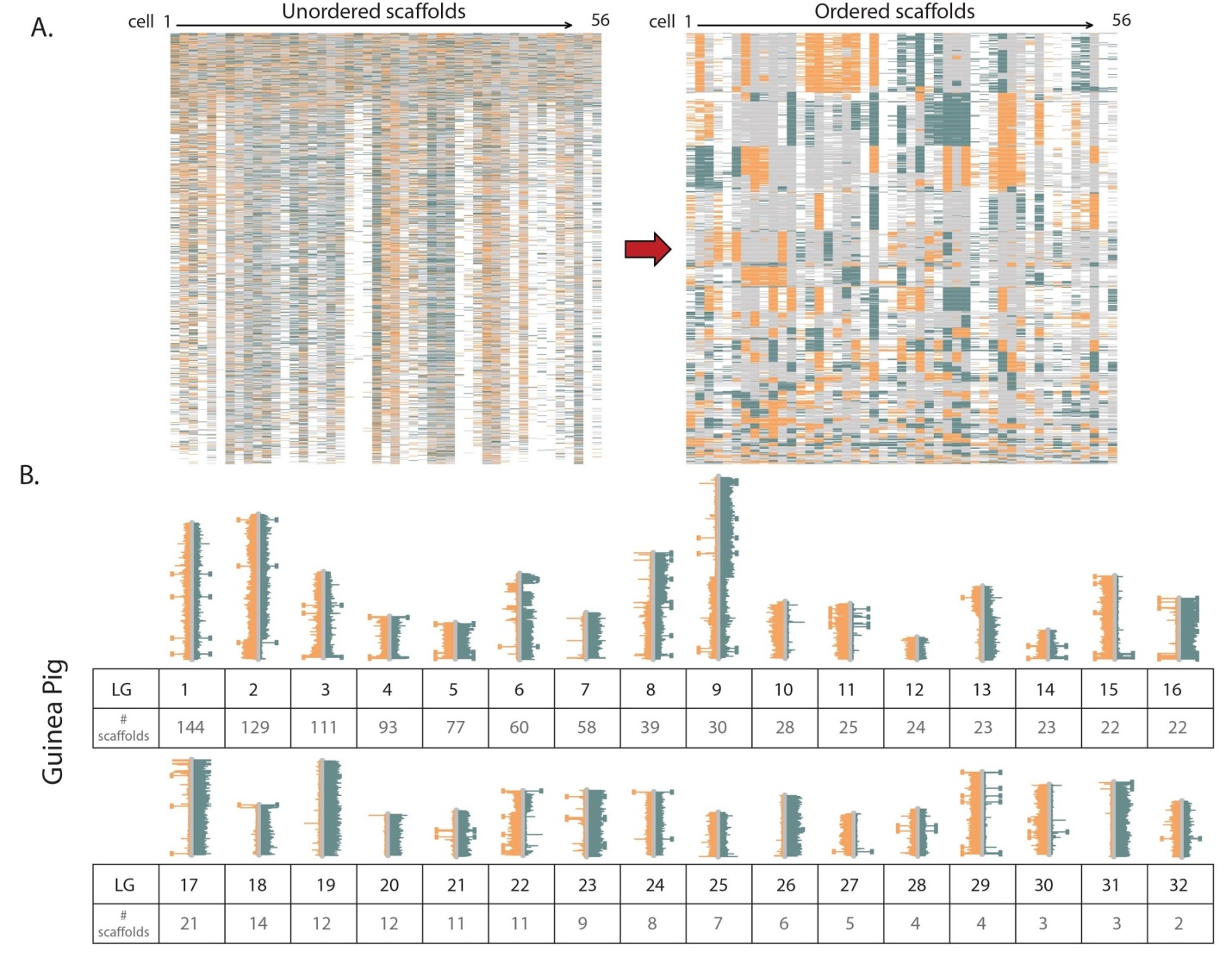
**

**Supplementary Figure 4: Guinea pig assembly made from non-contiguous scaffolds based on Strand-seq data.**

A. Left panel shows Guinea pig scaffolds presented in the current assembly order. Right panel shows scaffolds after contiBAIT reordering B. Representative ideogram plot of a Guinea pig library after clustering and ordering scaffolds. Each linkage group is represented by a certain number of scaffolds. Chromosomes with WW, WC and CC inheritance patterns are observed in this library. Changes in strand state represent SCE events.

**Supplementary Figure 5:**


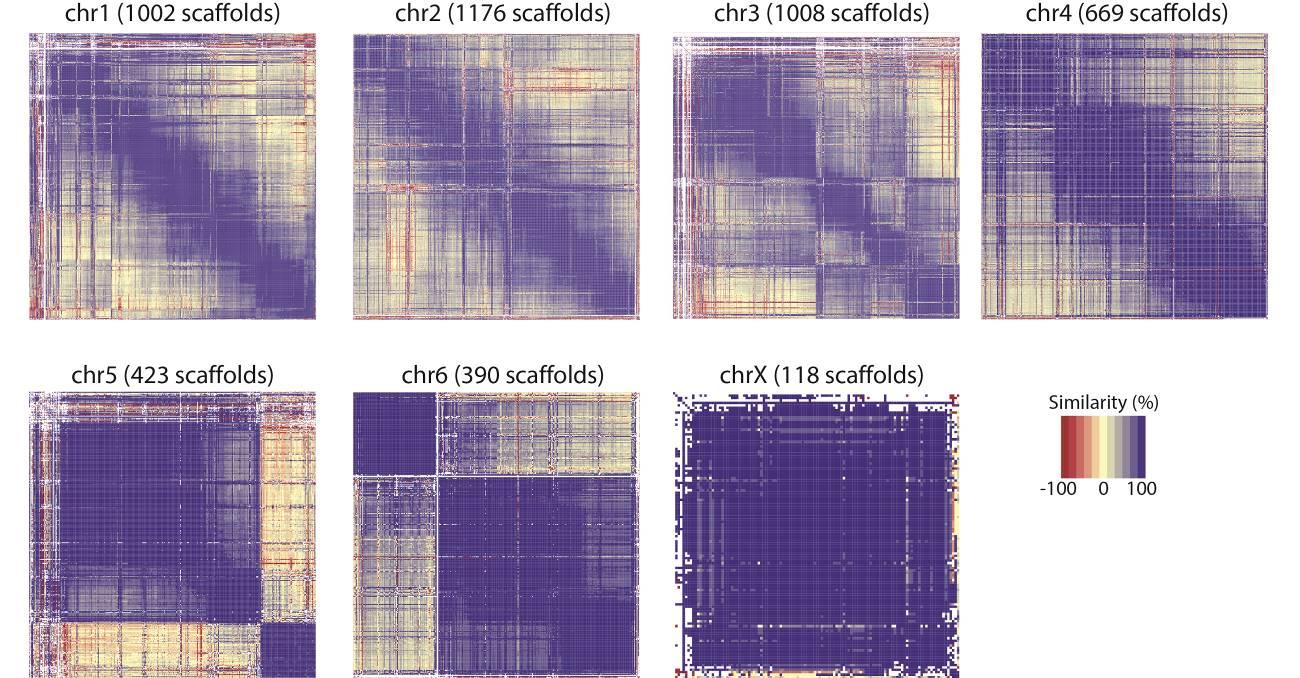


**Supplementary Figure 5: Tasmanian devil scaffolds ordered within chromosomes.** While the Tasmanian devil assembly consists of 35,974 scaffolds, most of these fragments have been located to specific chromosomes within the assembly (6 autosomes and an X chromosome). Heat maps show the relative order of these fragments within this assembly. A total of 4,786 scaffolds were clustered, which represents 90.4 % (2,869.1 Mb) of the Tasmanian Devil assembly.

**Supplementary Figure 6**

**
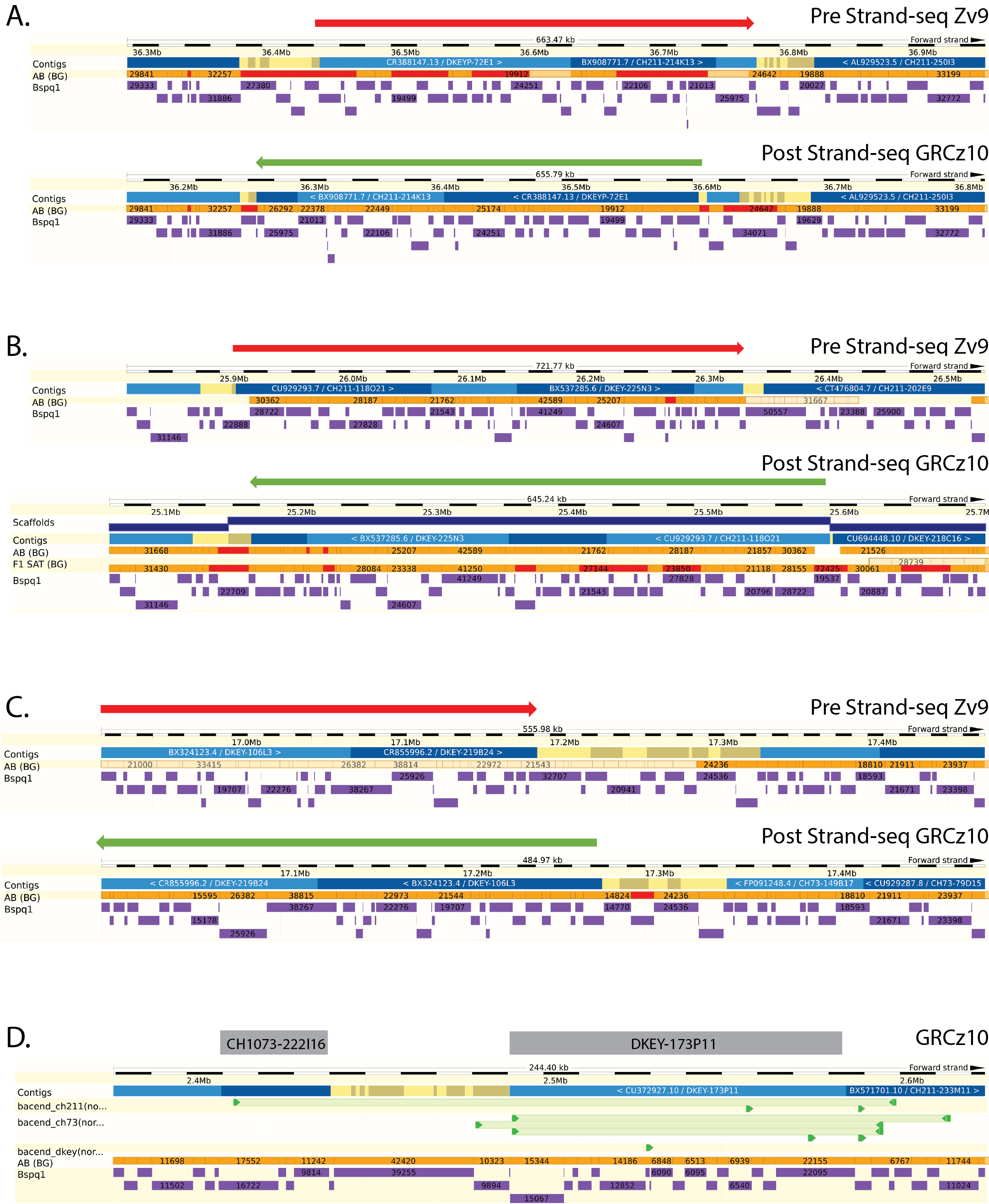
**

**Supplementary Figure 6: Characteristic examples of gEVAL screenshots showing Strand-seq and optical mapping data plotted on successive assemblies of the Zebrafish genome.**

A. An inversion of four-clone-contig (DKEYP-72E1 and CH211-214K13) on chromosome 3 suggested by Strand-seq data was confirmed by optical mapping data (shown in orange, *in silico* cuts in purple). The alignment vastly improves after reorientation.

B. An inversion of a four-clone-contig (CH211-188O21, DKEYP-121E10, DKEY-225N3 and CH73-69C10) on chromosome 24 as suggested by Strand-seq data. In the pre-Strand-seq version, no alignment could be achieved, after reorientation optical maps from two different strains align well against the updated reference. The alignment discordances are mostly caused by inter-strain variation.

C. An inversion of a two-clone-contig (DKEY-106L3 and DKEY-219B24) on chromosome 10 as suggested by Strand-seq data. The alignment between optical maps and genome reference vastly improves after reorientation.

D. Positioning of the previously unplaced clone CH1073-222I16 next to DKEY-173P11 on chromosome 13 as suggested by Strand-seq data. This placement is confirmed by optical mapping data and clone end alignments (shown in green).

gEVAL displays optical mapping data in orange and the respective *in silico* cuts within the assembly in purple. Within the optical mapping feature track, orange colouring indicates alignment, red discordance and light yellow complete lack of alignment. The zebrafish assembly versions can be found in gEVAL at <https://geval.sanger.ac.uk/AGP_zfish/Info/Index> and <https://geval.sanger.ac.uk/Zfish_GRCz10/Info/Index>.

**Supplementary Figure 7**

**
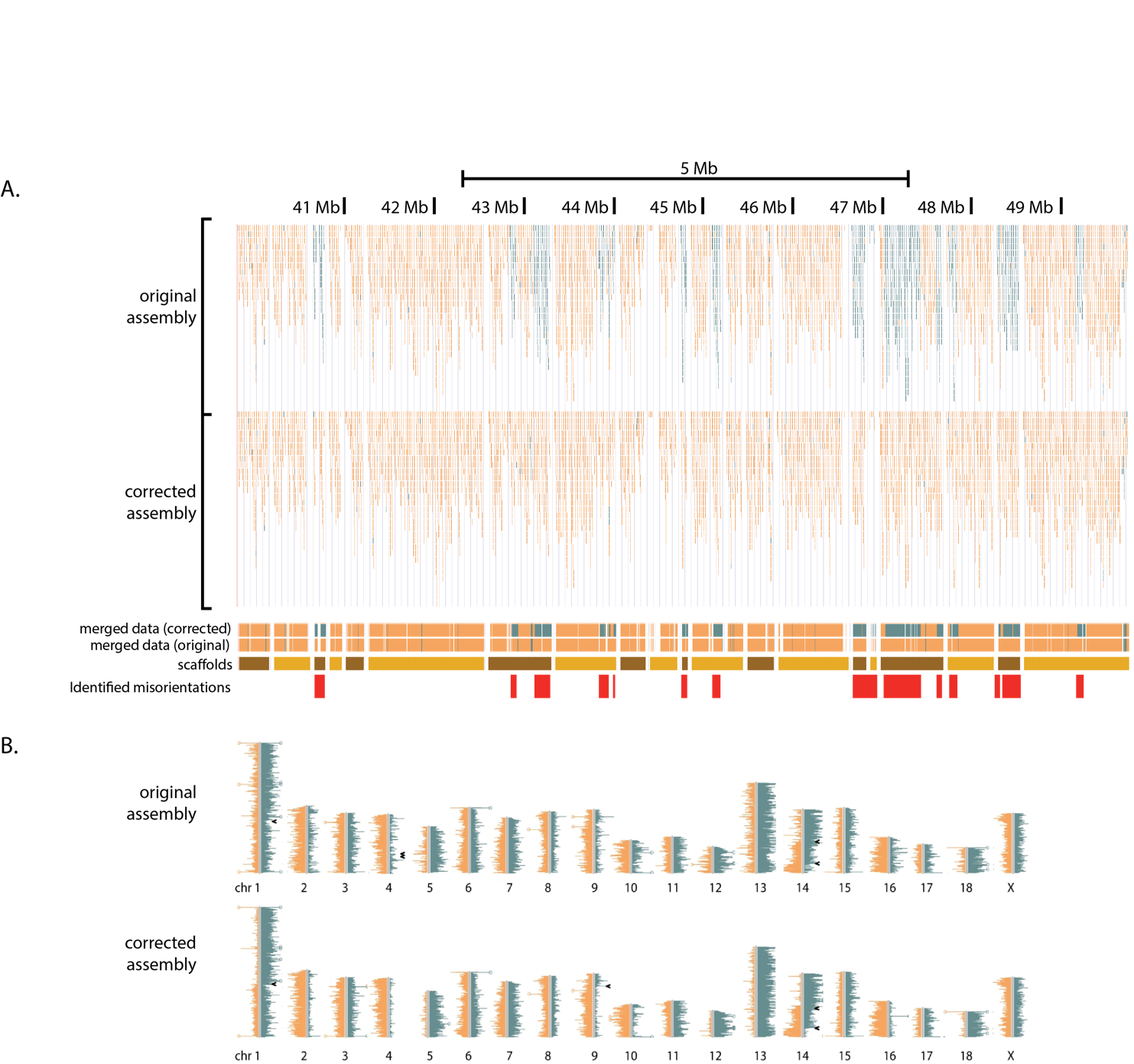
**

**Supplementary Figure 7: Correcting the orientation of scaffolds in the pig assembly.**

A. 17.8 % of the pig assembly was identified as misoriented. By reverse complementing regions on misorientation, we have corrected this assembly with Strand-seq data. BED plots show read distribution and directionality from a UCSC genome browser screenshot. Red boxes represent locations identified at misorients. Note these events occupy both entire scaffolds, or portions of scaffolds (suggesting errors within contiguous sequence). B. BAIT ideograms for a single cell generated before and after correction of the pig assembly. The template strand inheritance pattern cannot be distinguished in the original assembly due to high prevalence of WC states, but is resolved after reorientation. Arrowheads indicate locations of sister chromatid exchange events.
