# Supplementary figures and images for: Construction of Whole Genomes from Scaffolds Using Single Cell Strand-Seq Data
Source: Int J Mol Sci. 2021 Mar 31;22(7):3617. doi: 10.3390/ijms22073617 (PMC8037727; doi:10.3390/ijms22073617)

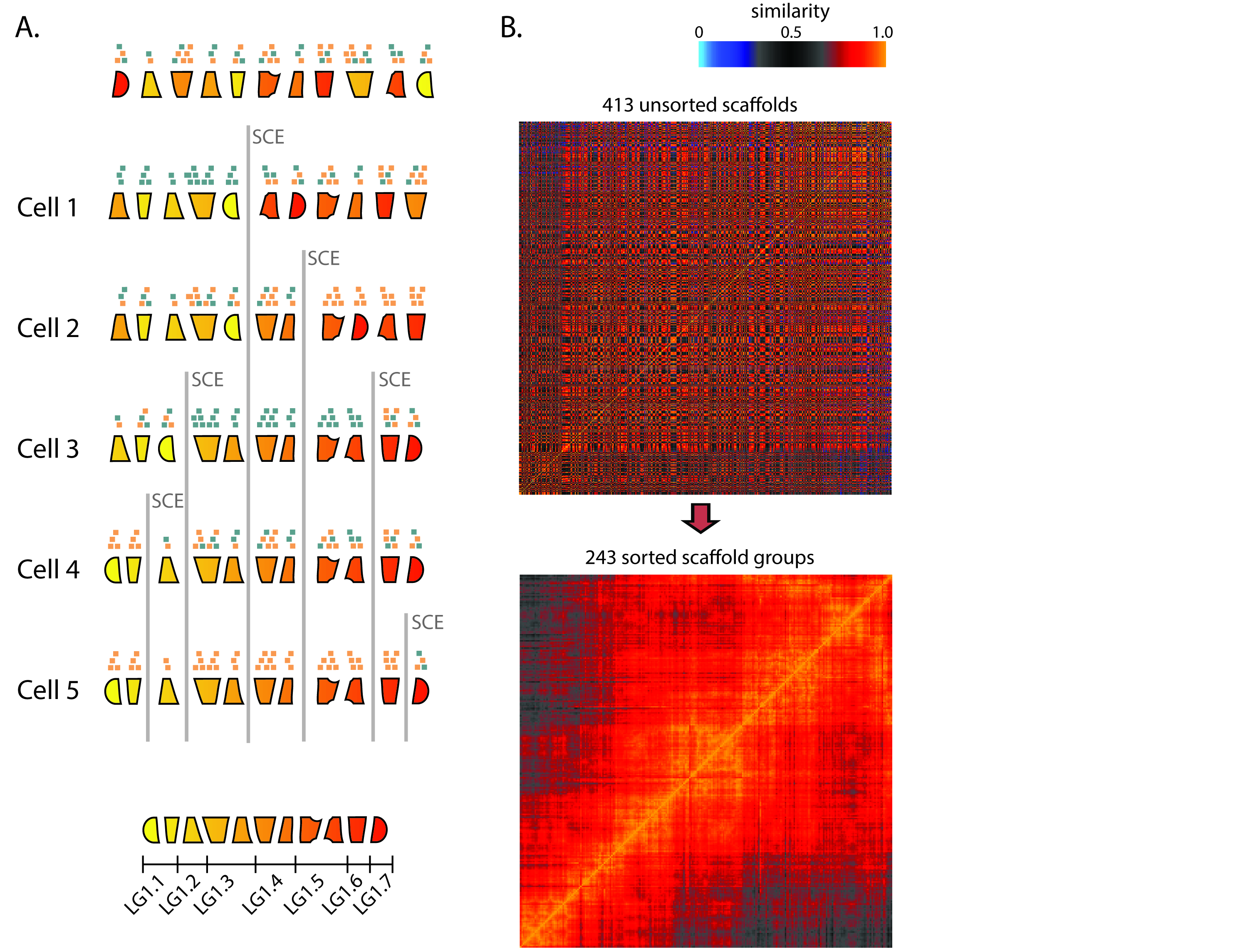

Supplement: Supplementary file 1 [file ijms-22-03617-s001.zip › Hills et al - Supplementary Information/SI Figure 1.tif]

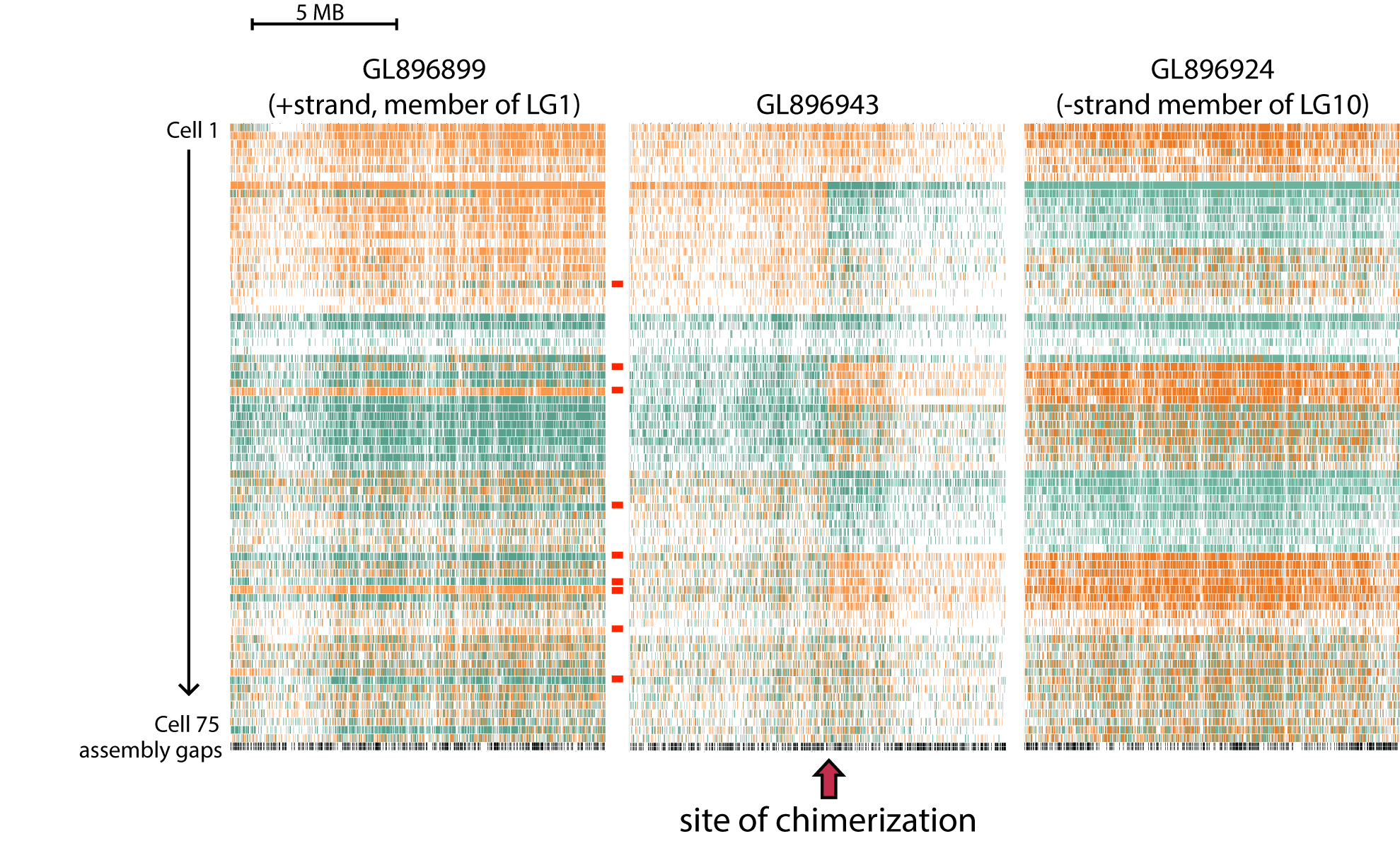

Supplement: Supplementary file 1 [file ijms-22-03617-s001.zip › Hills et al - Supplementary Information/SI Figure 2.tif]

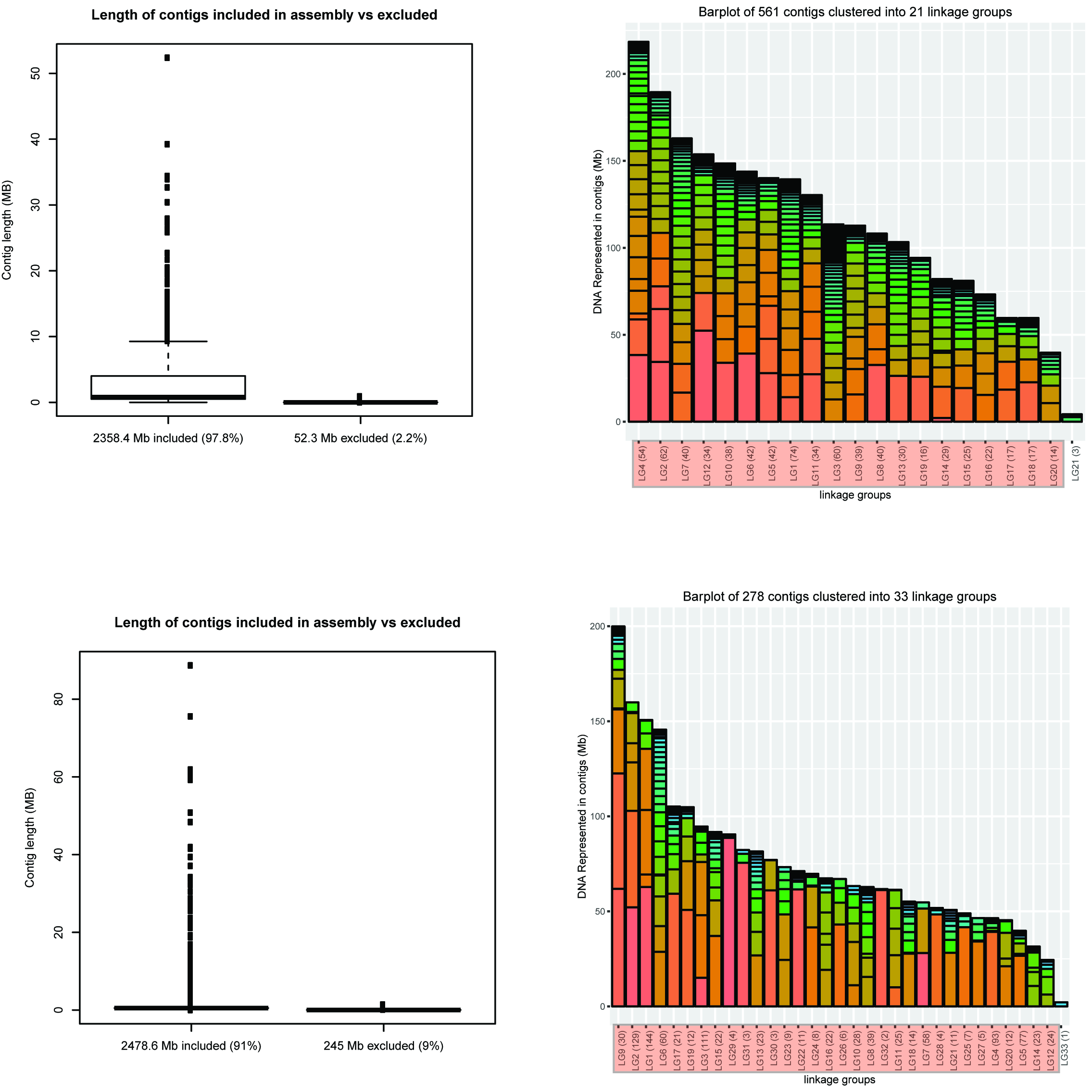

Supplement: Supplementary file 1 [file ijms-22-03617-s001.zip › Hills et al - Supplementary Information/SI Figure 3.tif]

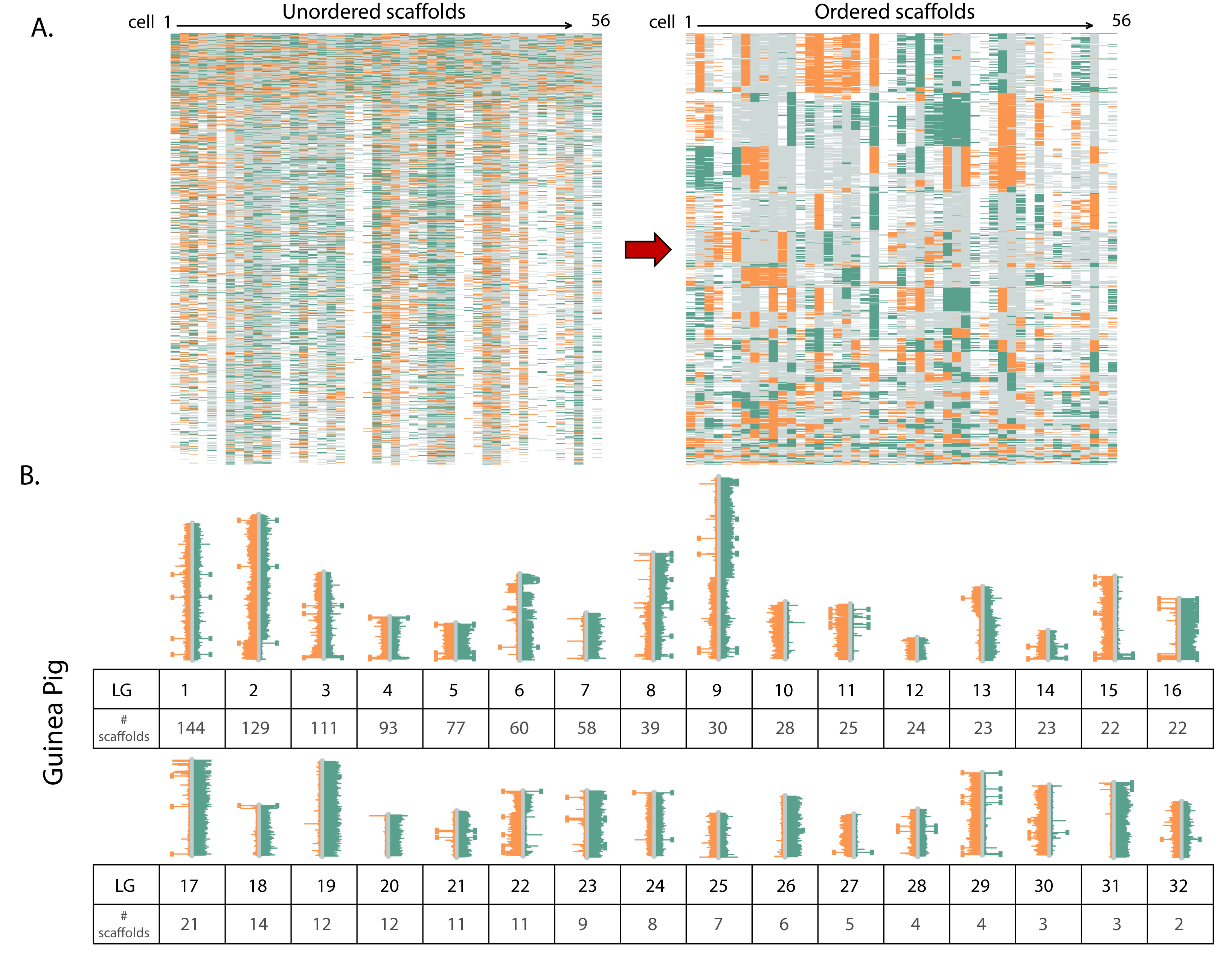

Supplement: Supplementary file 1 [file ijms-22-03617-s001.zip › Hills et al - Supplementary Information/SI Figure 4.tif]

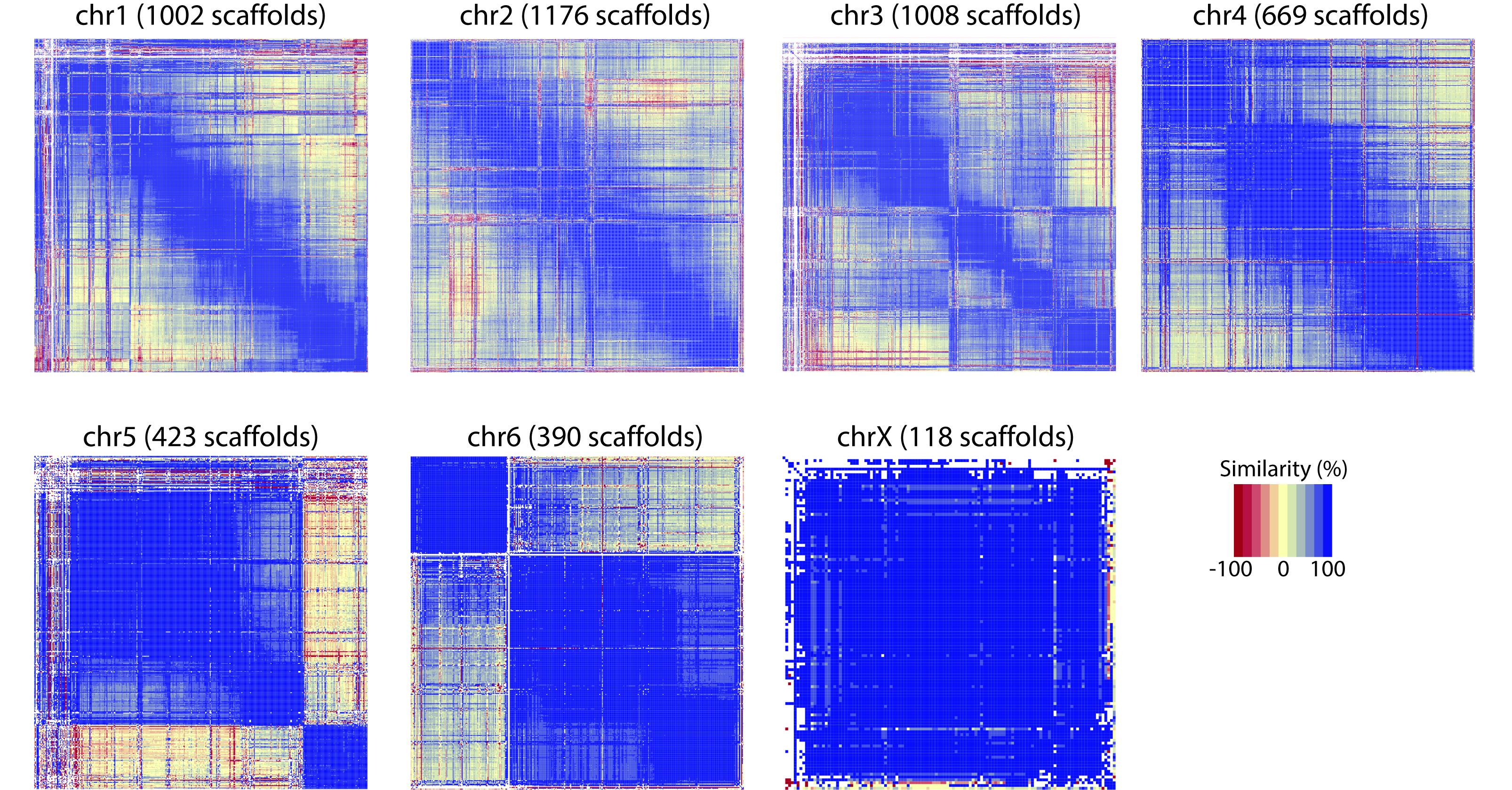

Supplement: Supplementary file 1 [file ijms-22-03617-s001.zip › Hills et al - Supplementary Information/SI Figure 5.tif]

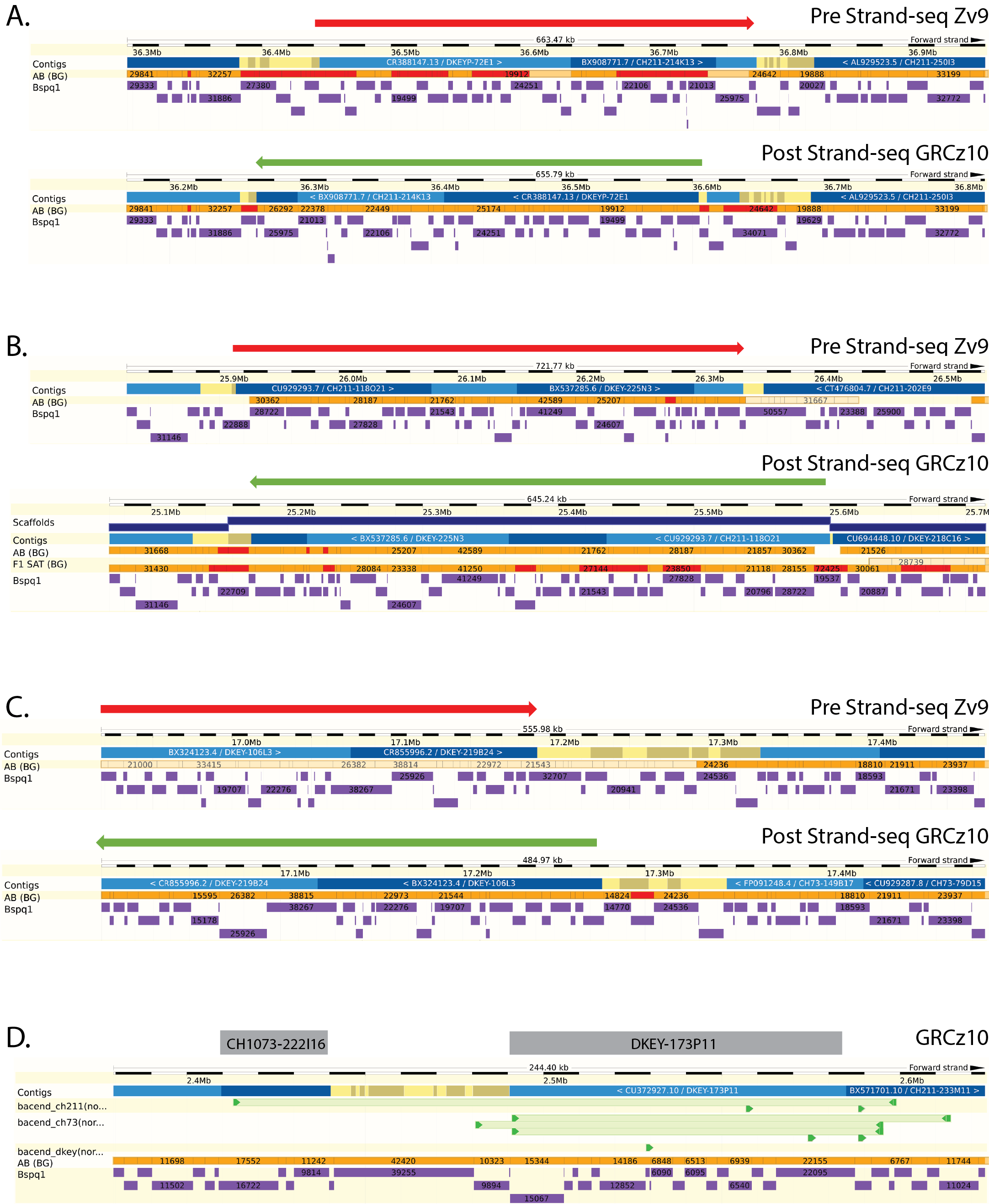

Supplement: Supplementary file 1 [file ijms-22-03617-s001.zip › Hills et al - Supplementary Information/SI Figure 6.png]

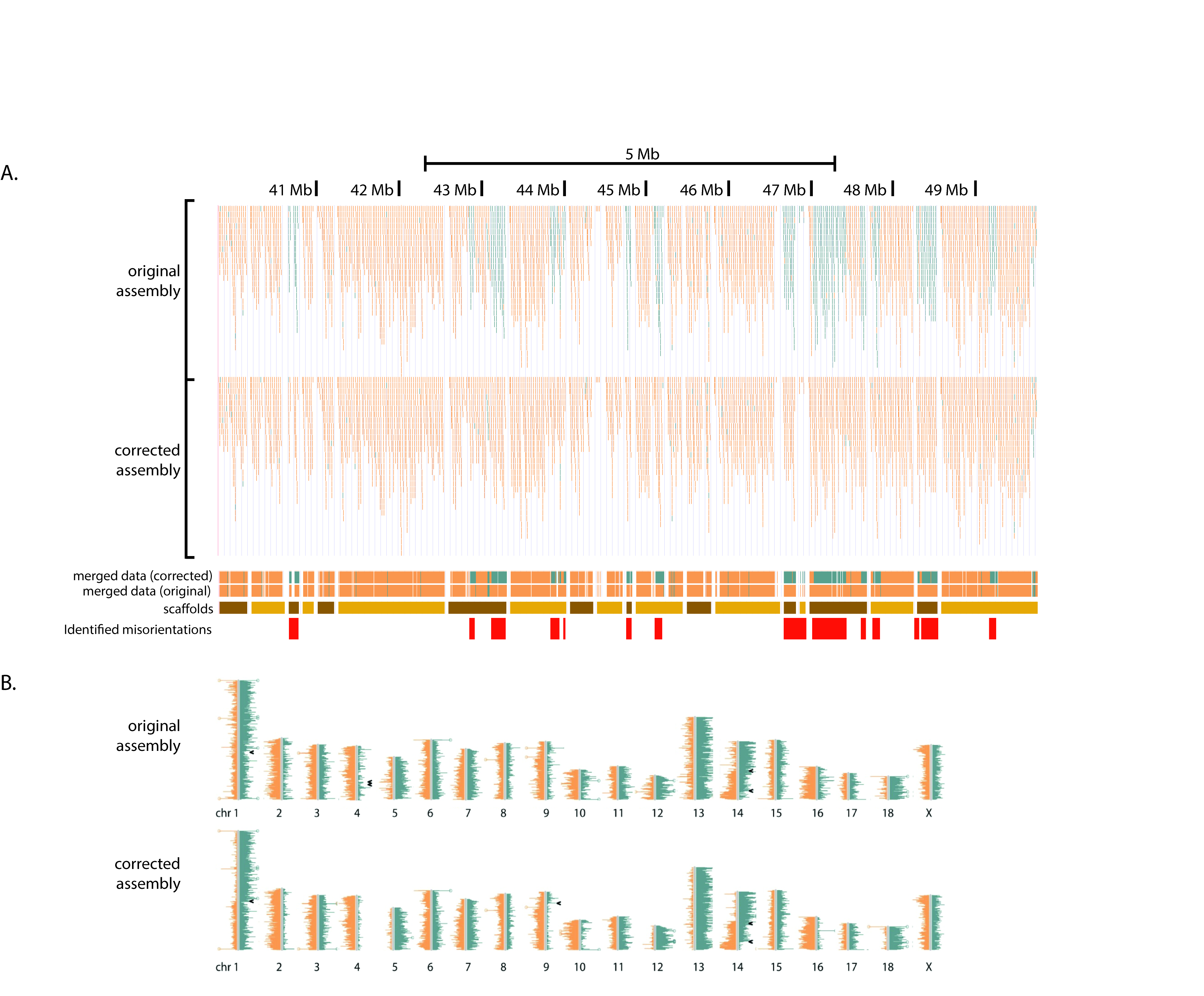

Supplement: Supplementary file 1 [file ijms-22-03617-s001.zip › Hills et al - Supplementary Information/SI Figure 7.tif]
